# Multiwavelength, Ultranarrow Line Width Emission from Fiber-Capillary-Integrated Colloidal Quantum Well Lasers

**DOI:** 10.1021/acsnano.5c04750

**Published:** 2025-07-21

**Authors:** Rui Duan, Yi Tian Thung, Yichen He, Guodan Wei, Yuan Wang, Lian Xiao, Zitong Zhang, Tianhua Ren, Lin Zhang, Van Duong Ta, Handong Sun

**Affiliations:** 1 Institute of Applied Physics and Materials Engineering, 59193 University of Macau, Macao SAR 999078, China; 2 Division of Physics and Applied Physics, School of Physical and Mathematical Sciences, 54761 Nanyang Technological University, 21 Nanyang Link, Singapore 637371, Singapore; 3 School of Precision Instruments and Optoelectronics Engineering, 12605 Tianjin University, Tianjin 300072, China; 4 Institute of Semiconductors, 514144 Guangdong Academy of Sciences, Guangzhou, Guangdong 510650, China; 5 Department of Optical Devices, 344748 Le Quy Don Technical University, Hanoi 100000, Vietnam

**Keywords:** laser source, colloidal quantum wells, multiwavelength, narrow line width, integrated device

## Abstract

Multiwavelength laser sources are indispensable in the development of quantum information, laser lighting, and laser display technologies. However, conventional multiwavelength lasers are costly, cumbersome, and intricate, limiting their adaptability across various applications. To overcome these challenges, we propose an integrated multiwavelength laser source based on solution-processed colloidal quantum wells (CQWs). Through precise control over size, elemental doping, and the design of heterostructures, we have engineered CQWs that exhibit ultralow stimulated emission thresholds across red, orange, yellow, and green wavelengths. CQW-based multiwavelength laser source delivers single-mode lasing with narrow line widths below 50 pm. Importantly, the laser structure is optimized via optical fiber coupling strategies, eliminating the need for complex micromanipulation procedures. Furthermore, we provide a preliminary demonstration of the applications of CQW–optical fiber lasers in automobile exhaust sensing, confirming their great promise for further development. These impressive specifications for colloidal semiconductor lasers highlight their strong potential as a powerful platform for next-generation visible light laser technologies.

## Graphical abstract

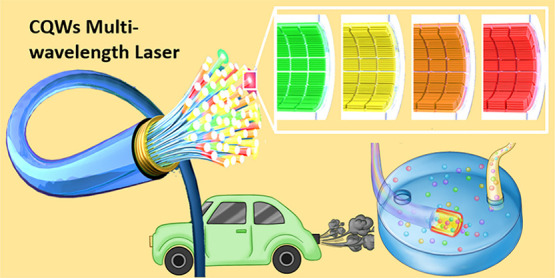

## Introduction

Multiwavelength coherent light sources are pivotal in advancing modern physics and optoelectronic technologies.^1–5^ Recent studies have highlighted their critical role establishing fully and synchronously connected multiuser quantum networks.^6^ Moreover, multiwavelength laser sources have been proven to significantly boost the sensitivity of quantum computing.^7–9^ These narrow line width multicolor laser sources are also essential in laser lighting and display technologies. Compared to traditional light-emitting diode and liquid crystal displays, lasers provide luminance that surpasses them by at least 2 orders of magnitude, offer a lifespan 10 to 20 times greater, and deliver superior color gamut and resolution.^10–12^ Despite these compelling advantages, the deployment of multiwavelength laser sources still faces considerable challenges, including the complexity of the necessary components and complex manufacturing processes. Further research is needed to reduce costs and achieve miniaturized designs.

The key to overcoming these challenges hinges on the meticulous optimization of the gain medium and the laser cavity. The gain medium is crucial in defining the emission wavelength, while at the same time the cavity plays a pivotal role in facilitating the integration of the laser system. Colloidal quantum wells (CQWs), also known as nanoplatelets, are increasingly recognized as some of the most promising candidates for laser gain mediums. They offer substantial advantages, including large gain cross sections,^13–17^ suppressed inhomogeneous broadening,^15,18^ and high modal gain coefficients.^13,19,20^ Essentially, CQWs are two-dimensional (2D) nanosheet with thickness quantized in atomic layers, which allows for precise control over their emission wavelengths by adjusting the thickness and material composition.^21–23^ Distinct from the complex and expensive manufacturing techniques required for traditional semiconductors, CQWs can be synthesized using solution-based processes that are both more economical and simpler to execute.^24–26^ Crucially, solution processability facilitates the integration of these CQWs into various laser resonators and lays the foundation for cost-effective and scalable manufacturing technologies for micro- and nanolasers, such as spin-coating, inkjet printing, nanoimprinting, and self-assembly.^27–34^ Trials with various types of CQW-based microlasers have yielded promising results, featuring low thresholds and high-quality spectral outputs.^13,20,34–40^ Despite these considerable advances, multiwavelength laser sources based on CQWs have yet to be explored. Additionally, the integration of colloidal nanocrystal materials into commercial components remains challenging, significantly hindering the practical progression of next-generation solution-processable laser technologies.

In this study, we propose a CQW-activated multiwavelength laser source, combining the unique advantages of CQWs with the extensive applicability of fiber technology. CQWs spontaneously deposit at the ends of capillary tubes to form laser emitters and are stimulated through fiber coupling, thereby eliminating the need for complex microfocusing systems. Compared to conventional free-space optical pumping, our CQW laser system provides enhanced integration and considerable flexibility, facilitating both pumping and signal collection. By meticulously adjusting the size and elemental composition of CQWs, the proposed CQW laser achieves a high-quality factor (>10^4^) and low-threshold output for single-mode lasing across the green, yellow, orange, and red spectral bands. Furthermore, the applications of CQW–optical fiber lasers as automobile exhaust sensors have been successfully demonstrated. We believe that this study will advance the integration of optoelectronic devices based on colloidal luminescent materials, contribute to the development of next-generation visible light laser technologies, and provide essential support for sensing, modern communications, display, and other optoelectronic applications.

## Optical Characterization of Four CQWs with Different Emission Bands

By combining size tuning, elemental doping, and heterostructure engineering strategies, we successfully synthesized red-emitting CdSe/Cd_1–*x*_Zn_*x*_S core/shell CQWs, orange-emitting CdSe/Cd_1–*x*_Zn_*x*_S core/thin-shell CQWs, yellow-emitting CdSeS/Cd_1–*x*_Zn_*x*_S core/shell CQWs, and green-emitting bromide ligand-capped CdSe CQWs using the hot-injection method. Four types of CQWs were synthesized based on bare CdSe (or CdSeS) CQWs with a uniform thickness of four monolayers (4 ML), as detailed in the Experimental Section of the Supporting Information. For red-emitting core/shell CQWs, the Cd_1–*x*_Zn_*x*_S alloy shell provides robust type-I confinement to inhibit exciton interaction with surface defects and reduce the dissipative biexciton Auger recombination rate. These factors collectively improve the photoluminescence quantum yield (PLQY) and gain properties. The synthesized CQWs display a photoluminescence (PL) emission full-width at half-maximum (fwhm) of ∼24 nm, with a PLQY measured at up to ∼0.96 (Figure 1a). Transmission electron microscopy (TEM) images reveal that the synthesized CQWs have a uniform rectangular shape (Figure S1). To assess the gain properties of these core/shell CQWs, amplified spontaneous emission (ASE) measurements were performed by using the stripe geometry method. A 5 ns, 20 Hz pulsed laser was used for excitation in all ASE and lasing experiments unless otherwise noted. As illustrated in Figure 1b, an increasingly narrower emission band emerged on the red side of the spontaneous emission band with rising nanosecond pump energy density, signaling the onset of ASE. The emission intensity’s dependence on pump strength confirms that the ASE threshold for the synthesized CQWs is remarkably low at only 18 μJ cm^–2^.

**Figure 1:**
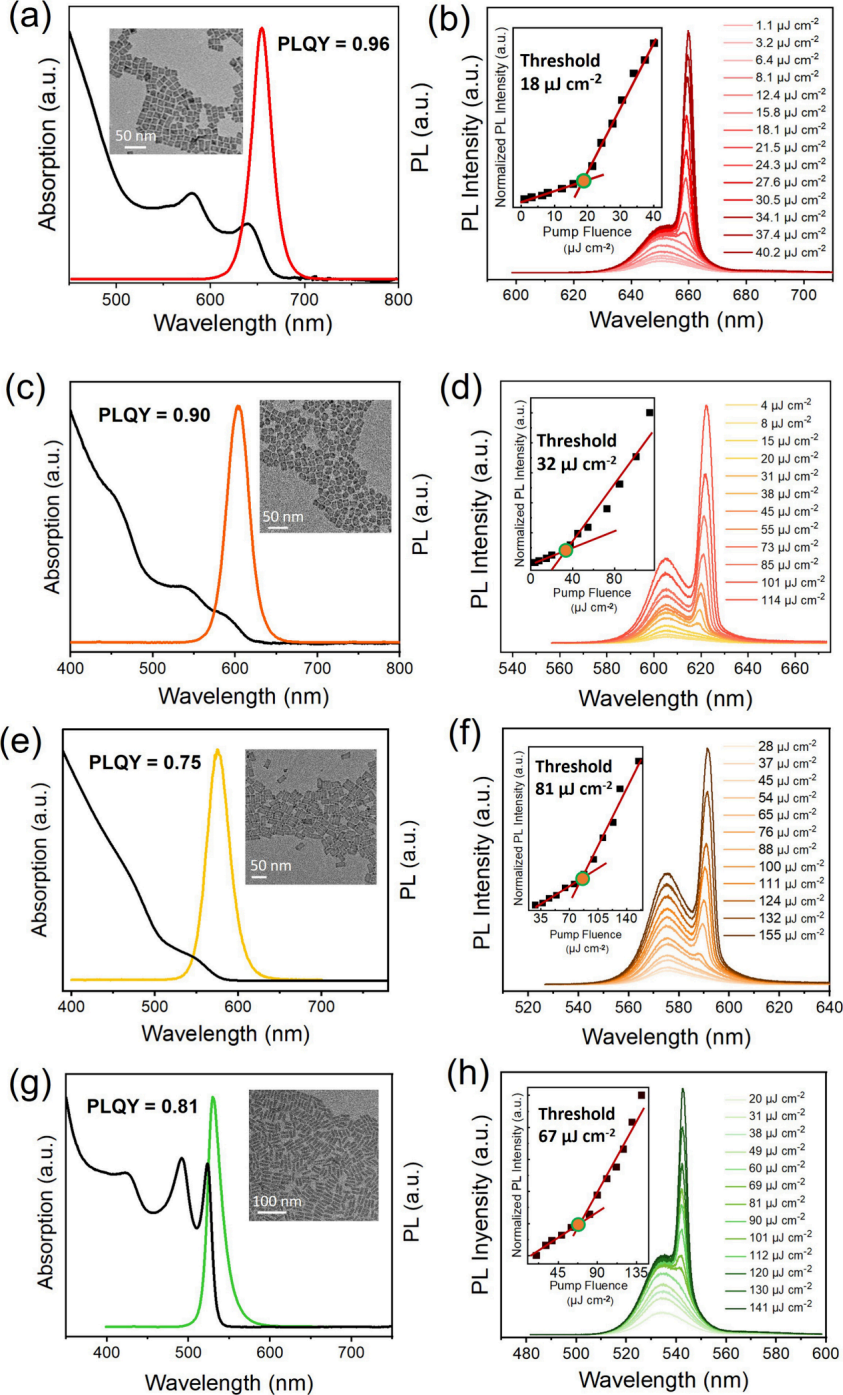
Optical characterization of four CQWs with different emission bands. (a, c, e, g) Absorbance and PL spectra of the as-synthesized (a) red-emitting CdSe/CdS@Cd_1–*x*_Zn_*x*_S core/buffer shell@graded-shell CQWs, (c) orange-emitting CdSe/Cd_1–*x*_Zn_*x*_S core/thin-shell CQWs, (e) yellow-emitting CdSeS/Cd_1–*x*_Zn_*x*_S core/shell CQWs, and (g) green-emitting bromide ligand-capped CdSe CQWs each accompanied by their PLQY at room temperature. The inset shows TEM image of the CQWs. (b, d, f, h) ASE measurement of four films prepared using red, orange, yellow, and green CQWs under nanosecond pulse laser excitation. The insets show the normalized emission intensity as a function of pump fluence.

For orange-emitting CQWs, a modified shell synthesis protocol is implemented for the growth of a thin Cd_*x*_Zn_1–*x*_S shell on bare 4 ML of CdSe CQWs. The resulting heterostructure CQWs exhibit an orange PL emission centered at 603.4 nm with a PLQY of ∼0.90, and the TEM image is shown in the inset of Figure 1c. The thin shell growth induces a lesser degree of redshift when compared to the established shell growth protocol. The shell passivation confers a high PLQY. The emission line width is slightly broader compared to red-emitting core/shell CQWs grown using the contemporary protocol. The greater degree of inhomogeneous broadening is attributed to the modified synthesis protocol involving a lower growth temperature, which makes it more difficult to precisely control the reaction kinetics and, consequently, the thickness uniformity of the CQWs. Given that the optical properties of CQWs are highly sensitive to their thickness, such nonuniformity results in broadening of the PL spectral line width.

Yellow-emitting CQWs have a core composed of CdSe_1–*x*_S_*x*_ CQWs. Growing a Cd_1‑*x*_Zn*x*S shell on the CdSeS cores induces PL emission at ∼575 nm, and the PLQY was measured to be ∼0.75 (Figure 1e). From the TEM image, the as-synthesized CQWs present a rectangular shape with defined edges. The lower PLQY, compared to the CdSe/Cd_1‑*x*_ZnxS core/thin-shell CQWs, is likely due to the lower intrinsic emission efficiency of the CdSeS core and the increased lattice mismatch at the core/shell interface.^41^ Nevertheless, a PLQY of 0.75 achieved in the yellow-emitting region clearly demonstrates the effectiveness of our core–shell design strategy.

Bromide ligand-capped CdSe CQWs exhibit longer emission and absorption wavelengths than their counterparts capped with traditional carboxylate ligands.^42,43^ This redshift of optical features is attributed to the release of strain in the CQWs’ crystal structure and thus the subsequent extension of their lattice from the ligand exchange process, which coincides with our understanding of the quantum confinement effect. The PLQY of bromide ligand-capped CQWs is determined to be ∼0.80 (Figure 1g). From the TEM image, these CQWs exemplify high monodispersity and improved colloidal stability, evident from their lack of stacking and agglomeration. The exchange of surface passivating ligands also results in an increased luminescence, in tandem with the reports from the previous literature.^42,43^

We analyzed excitation-intensity-dependent ground-state bleaching (GSB) decay dynamics to compare the Auger lifetimes of four types of CQWs dispersed in hexane. As shown in Figure S2, the fast decay component becomes more prominent at higher pump fluence, indicating the involvement of biexcitons. After background subtraction based on established procedures,^44^ the decay curves were well fitted using single-exponential functions. The extracted Auger lifetimes for the CdSe/Cd_1‑*x*_Zn*x*S core/shell, CdSe/Cd_1‑*x*_ZnxS core/thin-shell, CdSeS/Cd_1‑*x*_ZnxS core/shell, and bromide ligand-capped CdSe CQWs are 242, 192, 160, and 67 ps, respectively (Figure S3), revealing a clear dependence on both core composition and shell architecture.

More importantly, under nanosecond pulse laser pumping, the ASE thresholds for red-, orange-, yellow-, and green-emitting CQWs were determined to be as low as ∼18, ∼32, ∼81, and ∼67 μJ/cm^2^, respectively, as depicted in Figure 1b,d,f,h. The low ASE thresholds provide an excellent foundation for constructing high-performance multiwavelength lasers based on CQWs.

## Multimode CQW Lasers Integrated into an Optical Fiber

Figure 2a presents a schematic of the multiwavelength laser based on CQWs. Leveraging capillary effects, the CQWs spontaneously form a laser emitter at the tip of the silica capillary. The capillary, made of fused silica, provides a broad optical transparency and low propagation loss. Its circular cross-section naturally supports whispering-gallery modes (WGMs), offering strong optical confinement and high-quality (*Q*) resonances. Furthermore, the capillary can be readily fused with multimode optical fibers, facilitating efficient light coupling and compact device integration while obviating the need for complex micromanipulation systems. The preparation method of the CQW–optical fiber lasers (CQW-OFLs) is summarized in Methods. The CQW-OFLs were tested by using a custom-built PL testing system, as shown in Figure S4. In our CQW-OFL devices, the CQWs are deposited onto the inner walls of the capillaries through solvent evaporation, leading to a largely random orientation of the quantum wells. As a result, the optical performance of the device exhibits negligible dependence on the aspect ratio of individual CQWs. Figure 2b depicts the spectral response of the CQW-OFLs under nanosecond pulsed laser pumping as a function of pump power. As the pump power increases, the characteristic peaks become sharper and more pronounced, indicating a transition from spontaneous to stimulated emission (Figure S5). The insets showcase microphotographs of the CQW-OFLs in both unexcited (i) and excited (ii) states, visually illustrating the effect of pump power on laser emission. At a pump power of ∼38 μW, a brilliant spot surpasses the brightness of spontaneous emission, providing visual evidence of laser generation. The lasing threshold for the CQW-OFLs was determined through curve fitting to be as low as ∼35 μW (Figure 2c). Three-dimensional finite-difference time-domain (3D-FDTD) simulations were conducted on the CQW-OFLs. Due to memory constraints, the diameter of the simulated CQW-OFLs was set to 5 μm, with the detailed 3D structure shown in Figure S6. The electric field distribution on both the *x*–*y* and *y*–*z* planes demonstrates that WGMs can be effectively excited within the CQW gain layer (Figure 2d,e). Additionally, Figures S7 and S8 further confirm that the laser type of CQW-OFLs is classified as WGM-based, as indicated by the number of laser modes and their size dependence.

**Figure 2:**
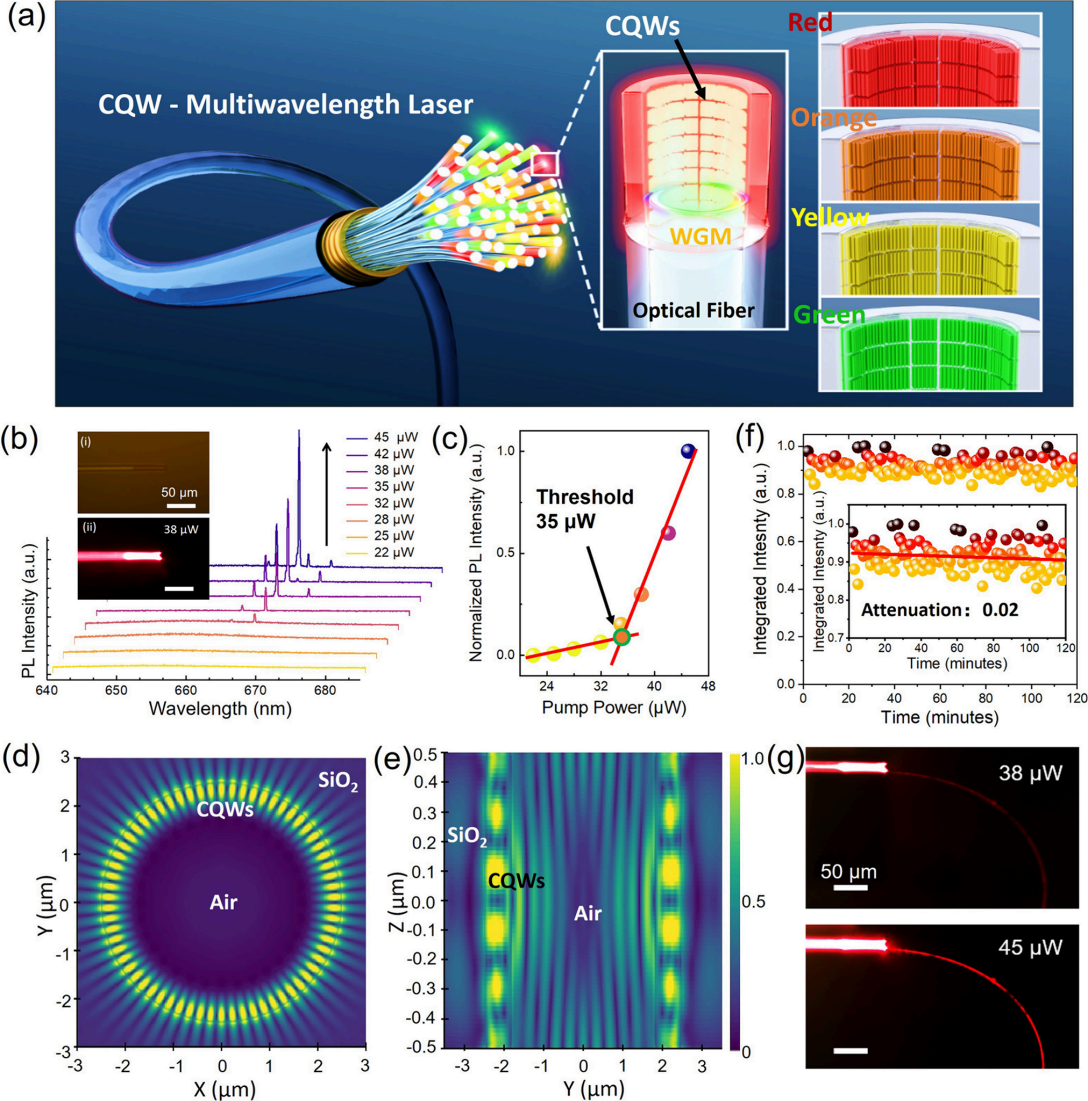
Multimode CQW lasers integrated into an optical fiber. (a) Schematic diagram of CQW-activated multiwavelength laser source. Driven by the synergistic effects of capillary forces and solvent evaporation, different types of CQWs spontaneously deposit at the ends of capillary tubes to form laser emitters. These emitters are activated through fiber coupling, eliminating the need for a complex micromanipulation system. The orientation of CQWs shown in the schematic illustration does not represent their actual orientation in the device. (b) Power-dependent emission spectra of CQW-OFLs with a diameter of ∼17 μm. Inset (i) displays the bright-field microscopic image of a CQW-OFL; inset (ii) shows the dark-field fluorescence image above the laser threshold. (c) Evolution of the normalized PL intensity of emission peaks as a function of pump power for a CQW-OFL. (d) Electric field distributions of CQW-OFLs simulated on the *x*–*y* plane. (e) Electric field distributions of CQW-OFLs simulated on the *y*–*z* plane. (f) Stability testing of CQW-OFLs. The tests were conducted at 5 times the laser threshold. (g) PL images showing light propagation in an ultrafine silica microfiber waveguide (diameter ∼500 nm) coupled to the end of a CQW-OFL at different pump powers.

Temporal stability is a crucial metric for evaluating microlaser performance. Therefore, we assessed the stability of the CQW-OFLs at a power level five times the laser threshold. As shown in Figure 2f, the laser output intensity exhibits an almost negligible decrease during a continuous 120 min testing period. This result indicates that the CQW-OFLs demonstrate promising temporal stability over a 2 h continuous operation, providing a foundation for reliable operation in future applications.

Finally, we report an intriguing coupling effect between the CQW-OFLs and an ultrafine silica microfiber waveguide, with a diameter of ∼500 nm. This microfiber is fabricated via flame-drawing from a standard single-mode fiber and exhibits a uniform diameter along its length. Its refractive index is ∼1.45, closely matching that of the silica capillary, which facilitates efficient mode matching between the waveguide and the capillary-based laser emitter. While such submicrometer-scale waveguide coupling typically requires nanoscale precision mechanical alignment, our CQW-OFL configuration simplifies the process by enabling passive evanescent coupling. The microfiber is sufficiently flexible to be gently wrapped around the capillary tip, naturally positioning itself within the evanescent field region of the WGMs (Figure S9). As shown in Figure 2g, efficient light transmission through the ultrafine waveguide was observed under both low and high pump powers.

## Multiwavelength Single-Mode CQW-OFLs

We aimed to achieve single-mode lasing by reducing the cavity size. Small diameter laser emitters were conveniently fabricated by using the flame pulling method (Figure S10). As illustrated in Figure 3a, three typical PL images of CQW-OFL emitters below, near, and above the threshold are showcased. The noticeable increase in the brightness of the emitters as the pump power indicates visually the onset of laser behavior. Figure 3b elaborates on the spectral characteristics of the laser under various pump powers. We noted a high-quality single-mode output with an fwhm of only 0.047 nm, corresponding to a lasing *Q*-factor (*Q* = λ/Δλ, where λ and Δλ are the lasing peak wavelength and lasing peak line width, respectively^28^) exceeding 14,000. The evolution of the output laser intensity with pump power established a laser threshold as low as ∼52 μW (Figure 3c). This excellent lasing *Q*-factor and low laser threshold highlight the significant potential of CQW-OFLs for single-mode output and applications.

**Figure 3:**
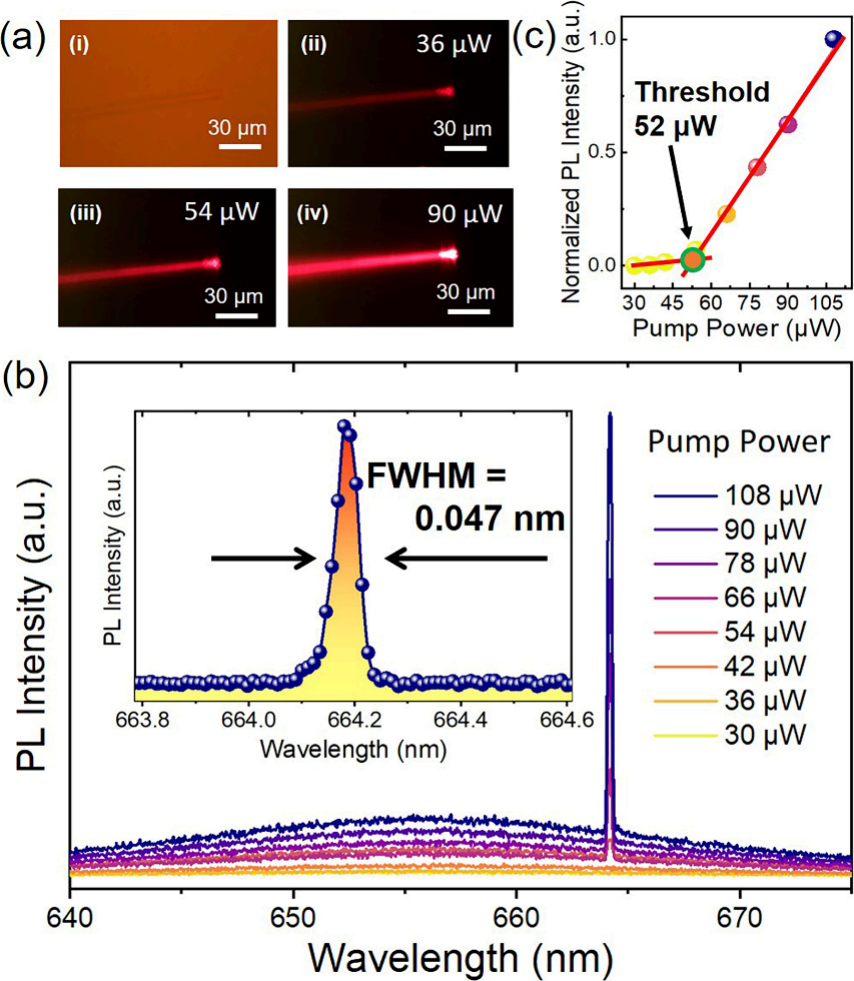
Single-mode, red-emitting CQW-OFLs. (a) Bright-field image of (i) a single-mode CQW-OFL, accompanied by typical PL images (ii) below, (iii) near, and (iv) above the laser threshold. (b) Spectral response of a single-mode CQW-OFL at different pump powers, with a measured fwhm of ∼0.047 nm. (c) Functional relationship between pump power and output laser intensity. The lasing threshold is determined by fitting to be ∼52 μW.

As mentioned in the first section, the emission wavelength can be precisely controlled by simply adjusting the size and elemental composition of colloidal CQWs. Since the proposed OFLs are not constrained by emission bands, we employed consistent laser fabrication procedures and excitation schemes to successfully achieve multiwavelength laser emissions (Figure 4). The emission bands for orange, yellow, and green single-mode lasers are demonstrated in Figure 4a,d,g. The insets display dark-field images of the CQW-OFLs above the laser threshold, highlighting the bright emission at the emitters. The laser thresholds for the three wavelengths were determined by fitting to be as low as 80, 181, and 164 μW (Figure 4b,e,h, respectively). The key performance parameters of the multiwavelength single-mode lasers, including emission band, fwhm, lasing *Q*-factor, threshold, and gain materials, are summarized in Table 1 and Table S1. Notably, each laser boasts a fwhm below 50 pm and a *Q*-factor exceeding 1 × 10^4^, regardless of the emission wavelength, confirming the remarkable capability of CQW-OFLs to generate high-quality monochromatic lasing.

**Figure 4:**
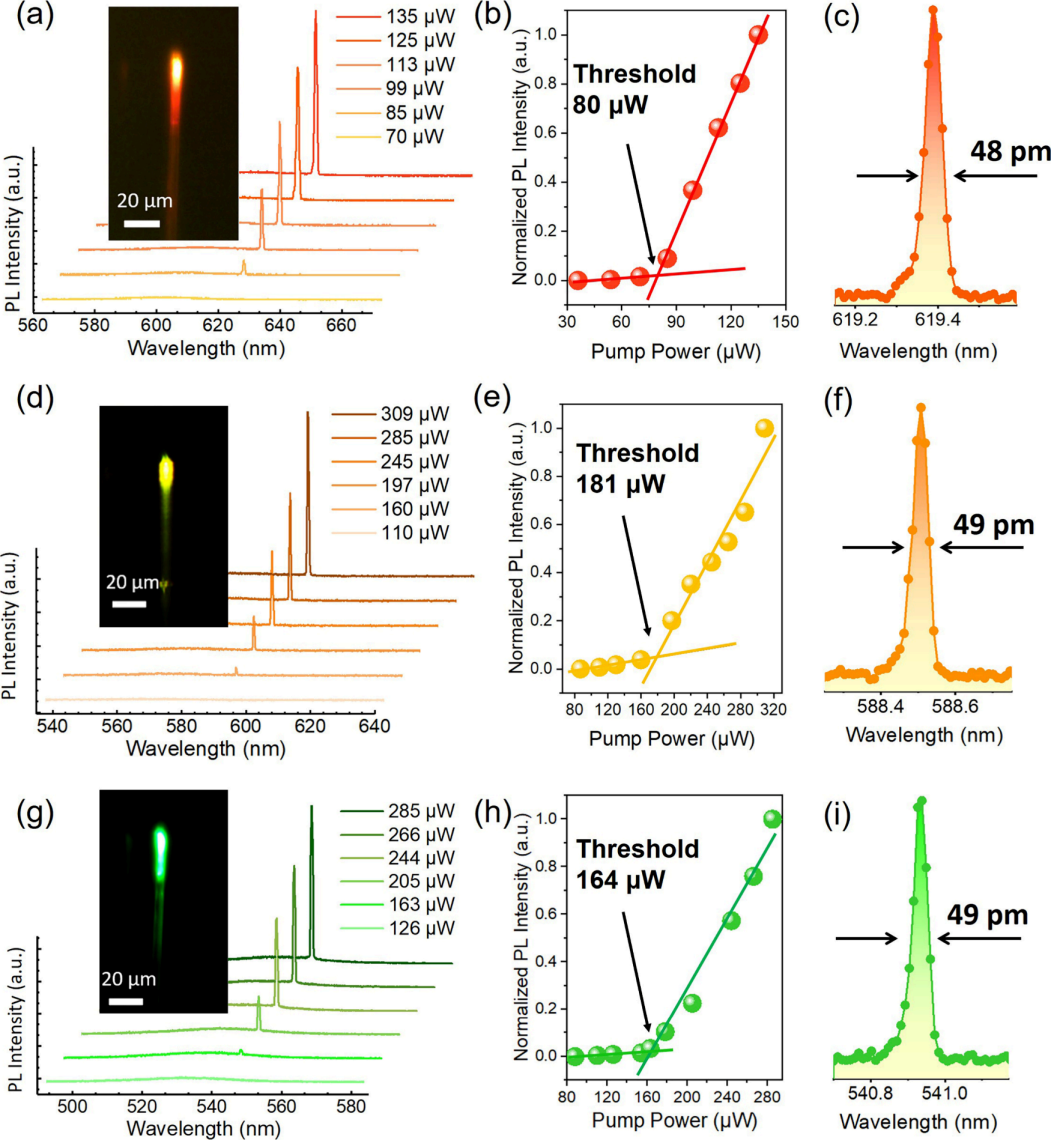
Multiwavelength single-mode CQW-OFLs. (a, d, g) Single-mode lasing emissions in the (a) orange, (d) yellow, and (g) green ranges are achieved using specially designed CQWs. The left insets display PL photographs of the multiwavelength lasers. (b, e, h) Evolution of the normalized PL intensity of emission peaks as a function of pump power for (b) orange, (e) yellow, and (h) green CQW-OFLs. (c, f, i) Detailed enlargements of the lasing peaks, illustrating the fwhm. Each laser achieves a fwhm of less than 50 pm.

**Table 1:** Lasing Features of CQW-OFLs^a^

emission band	fwhm (pm)	lasing *Q*-factor	threshold power (μW)	threshold of average power density (W cm^–2^)	gain materials
red	47	14000	52	1.69	CdSe/Cd_1–*x*_Zn_*x*_S C/S CQWs
orange	48	12900	80	2.61	CdSe/Cd_1__–*x*_Zn_*x*_S core/thin-shell CQWs
yellow	49	12000	181	5.90	CdSeS/Cd_1–x_Zn_*x*_S C/S CQWs
green	49	11000	164	5.35	bromide ligand-capped CdSe CQWs

a ns, nanosecond, C/S, core/shell.

## FRET-Enhanced Low-Threshold CQW-OFLs

FRET is a nonradiative energy transfer process facilitated by dipole–dipole interactions between donor and acceptor molecules.^45,46^ Indeed, when donor molecules are excited, they can nonradiatively transfer energy to acceptor molecules via FRET. This mechanism enhances the absorption of the acceptor, thus increasing the overall gain of the laser medium. In the realm of micro- and nanolasers, FRET has been utilized to broaden the gain spectrum and facilitate broadband lasing.^47,48^ Additionally, by adjusting the stoichiometry between donor and acceptor molecules, the efficiency of FRET can be fine-tuned to control the flow of excitons within complexes of colloidal materials.^46^ Consequently, by managing the efficiency of FRET, it is possible to modulate the overall density of the excited state, thereby optimizing the laser threshold for an improved performance.

The donor–acceptor pair we utilized consists of green-emitting bromide ligand-capped CdSe CQWs and red-emitting CdSe/Cd_1–*x*_Zn_*x*_S core/shell CQWs. Since the donor and acceptor CQWs have different PLQYs, to ensure the donor provides sufficient energy, we adjusted the concentration ratio of the quantum well solutions to donor:acceptor = 2.7:1. The adjusted mixed solution exhibits nearly equal emission peak intensities in the red and green wavebands, as depicted in Figure 5a. Furthermore, the absorption spectrum of the mixed solution perfectly matches the PL emission band of the donor. It is important to note that the ratio was determined in solution, where the average distances between individual CQWs typically exceed the Förster radius, thereby minimizing the FRET effects. However, after film formation or confinement within the microcavity, the reduced interdot distances enhance FRET, leading to a noticeable decrease in green emission intensity, as observed in range I of Figure 5b.

**Figure 5:**
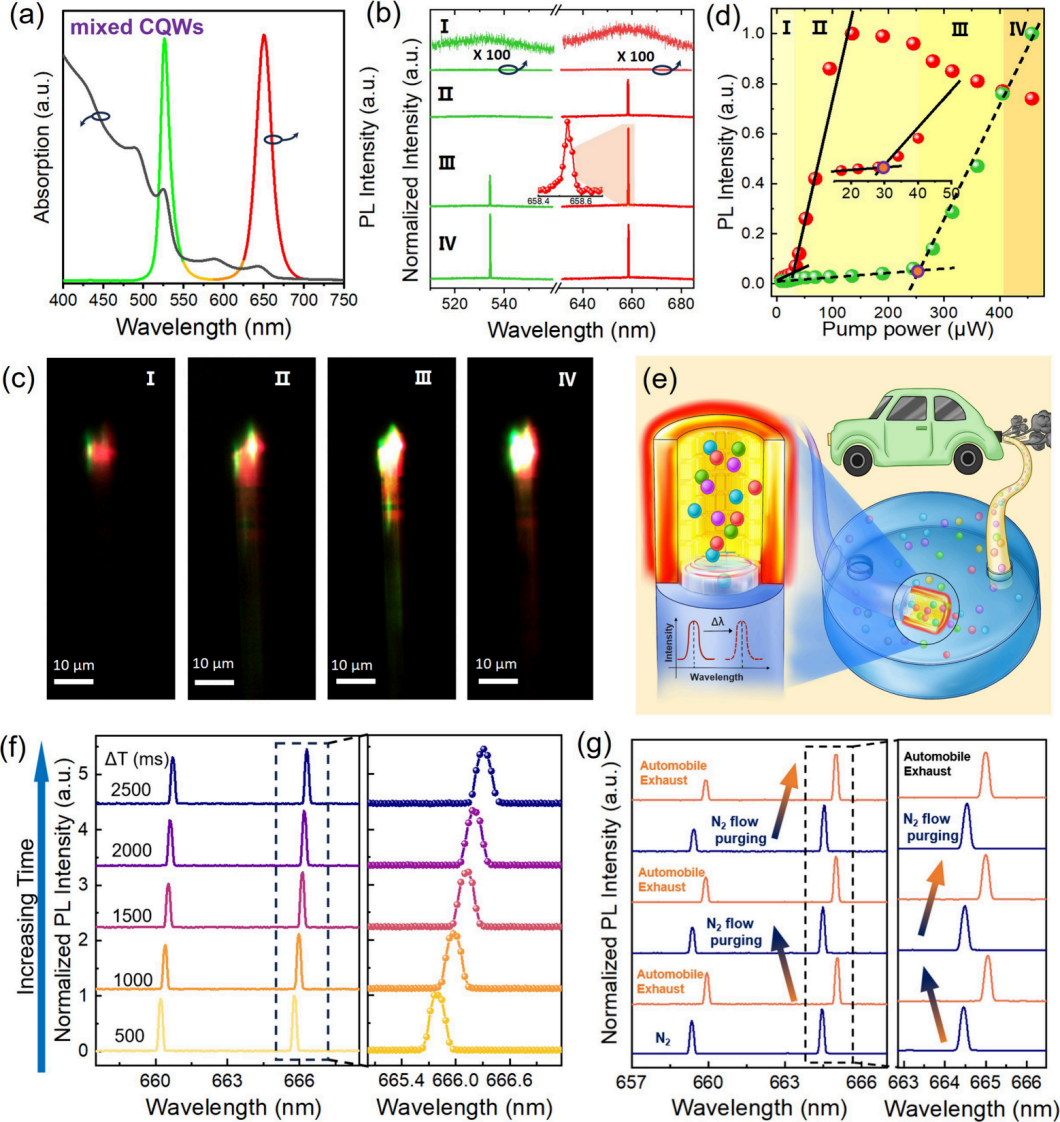
Low-threshold CQW-OFLs and automobile exhaust sensing. (a–d) FRET-enhanced low-threshold CQW-OFLs: (a) absorption and PL spectra of a mixed solution of green-emitting bromide ligand-capped CdSe CQWs (donor) and red-emitting CdSe/Cd_1–*x*_Zn_*x*_S core/shell CQWs (acceptor). (b) Four typical emission states in FRET-enhanced CQW-OFLs, arranged sequentially from top to bottom. (c) Dark-field fluorescence images corresponding to the four emission states shown in panel (b). (d) Evolution of the normalized PL intensity of the emission peaks as a function of pump power, divided into four sections from left to right. (e–g) Automobile exhaust sensing: (e) Schematic diagram of the mechanism for detecting automobile exhaust using CQW-OFL as a sensing probe. Inside the gas chamber, CQW-OFL interacts with exhaust molecules. Wavelength shifts serve as the indicative signal for exhaust detection. (f) Lasing spectra from CQW-OFLs acquired over the detection period during automobile exhaust monitoring. The inset provides a magnified view of the spectra near 666 nm. (g) Cyclic sensing test of CQW-OFLs for automobile exhaust and nitrogen.

Following the same preparation procedure as that before, the specifically designed donor and acceptor CQWs at a predetermined ratio were integrated into the OFLs. The PL decay curve of the composite system shows a significant reduction in the donor’s lifetime (Figure S11 and Table S2). This reduction is due to the additional decay pathway introduced by FRET, indicating effective energy transfer from the donor to the acceptor. The transfer efficiency, Φ_FRET_, is estimated based on the lifetimes as Φ_FRET_ = 1−τ/τ_0_, where τ and τ_0_ are the average lifetimes of the donor in the absence and presence of the acceptor, respectively. The calculated Φ_FRET_ is as high as 39%, with the donor’s lifetime decreasing from 1.36 to 0.83 ns.

Under nanosecond laser pumping, the spectra and PL images corresponding to four typical laser states were captured, as shown in Figure 5b,c. (I) In the initial state, with the pump power at only about 11 μW, only spontaneous emission was observed in two bands. Nonetheless, upon closer inspection, it was evident that the PL intensity in the red band was significantly higher than that in the green band. Given that we had balanced the mixed system of the two types of CQWs to the same intensity through concentration control, this disparity in PL intensity can be ascribed to the effects of FRET. (II) As the pump power was further increased to about 69 μW, red single-mode lasing emerged, while the green band continued to show only spontaneous emission. Furthermore, the PL image (Figure 5c-II) also displayed a marked contrast in luminous intensity between the two. (III) To turn on stimulated emission in the green band, the pump power is greatly increased to 405 μW. At this level, although the intensity of the red band laser was higher than in state (II), it had already exceeded its maximum emission intensity (as will be discussed subsequently). The PL image, as shown in Figure 5c-III, clearly displays a significant increase in the brightness of the green fluorescence. (IV) As the pump power was further increased, the intensity of the green band laser sharply intensified, while that of the red band notably declined. This confirmed that the donor CQWs were undergoing stimulated emission, thereby significantly suppressing the effects of FRET.

We now focus on the evolution of donor and acceptor PL emission intensities (*I*) as the pump power (*P*) varies, which is still divided into four corresponding regions, as shown in Figure 5d. In region I (*P* < ∼29 μW), both the donor and acceptor exhibit spontaneous emission with linear emission intensities. The results show that the FRET efficiency remains nearly constant at this stage, suggesting that FRET can occur smoothly even at low excitation powers. In region II (∼29 μW < *P* < ∼250 μW), a “knotting” pattern on the linear scale indicates the onset of stimulated emission in the acceptor CQWs, with the laser threshold identified as ∼29 μW. This value is significantly lower than the threshold observed for the core/shell CQW-OFLs, underscoring the critical role of FRET in markedly reducing the optical threshold. Indeed, as illustrated in Figure S12, ASE measurements on the film with the selected concentration ratio of donor and acceptor CQWs have shown a substantial decrease in the ASE threshold from 18.4 to 10.9 μJ cm^–2^, attributed to the effects of FRET. In region III (∼250 μW < *P* < ∼405 μW), the emission state of the donor underwent a transition, with laser behavior initiating at a pump power of ∼256 μW. At this juncture, the intensity of the donor’s emission increased rapidly with rising power. In region IV (*P* > ∼405 μW), the radiative intensity of the donor surpassed that of the acceptor. This phenomenon can be explained by two factors: first, the donor’s lasing behavior competes with the energy transfer process, significantly suppressing the FRET effect and causing the donor and acceptor to behave more like independent subensembles. Second, the pump power is high enough to induce thermal effects, which reduce the lasing performance of the acceptor.

The introduction of the FRET mechanism offers new possibilities for laser design and applications. Beyond lowering the laser threshold, FRET enables multiwavelength emission and intensity control in CQW lasers. We believe that this CQW-OFL technology incorporating FRET may drive the development of lower-cost and higher-performance laser systems.

## Automobile Exhaust Sensing

As industrialization accelerates and the number of motor vehicles increases, air pollution has become a global environmental issue. Automobile exhaust contains a variety of harmful substances, including carbon monoxide (CO), hydrocarbons (HC), nitrogen oxides (NO*_x_*), and a large amount of ultrafine particulate matter.^49^ In particular, UPMs in automobile exhaust are highly respirable, biologically active, and capable of penetrating deep into the lungs, bloodstream, and even the brain, posing significant threats to human health.^50,51^ Therefore, monitoring vehicle emissions is essential for safeguarding both the environment and public health. Here, we attempt to use CQW-OFLs as sensors to detect automobile emissions and verify their feasibility.

Figure 5e illustrates our schematic design. In this setup, CQW-OFLs act as the sensing probe, and automobile exhaust is pumped into the chamber through a window. It is well-known that when evanescent waves interact with molecules in the surrounding medium, the resonance wavelength shifts.^52^ Thus, this wavelength shift can serve as an indicator signal for exhaust detection. The CCD continuously recorded the spectrum at 500 ms intervals. Figure 5f displays the response spectrum of CQW-OFLs within a 2500 ms monitoring window. It is observed that the laser peak gradually shifts to red, reaching up to 530 pm over time. This result is understandable as the introduction of automobile exhaust gradually increases the environmental refractive index. Given that the refractive index of UPM (1.4–2.0) is significantly higher than that of gaseous pollutants (just above 1.0), the substantial presence of UPM in automobile exhaust is considered the primary contributor to the observed spectral shift. Furthermore, such high response sensitivity is inseparable from the high lasing *Q*-factor of the CQW-OFLs, which significantly amplifies minor disturbances. Notably, no significant change in laser intensity was observed throughout the entire monitoring window (Figure S13), indicating the system’s operational stability. Upon removal of the CQW-OFL sensor from the automobile exhaust and subsequent nitrogen purging, the lasing wavelength nearly returned to its original value, with only a minor residual redshift of 24 pm (Figure S14). This minor shift is speculated to be caused by the residual presence of a trace amount of UPM on the sensor surface. To further assess the sensor’s performance, a cyclic test was conducted by alternately exposing the CQW-OFL to automobile exhaust and nitrogen gas (Figure 5g). These results demonstrate the sensor’s preliminary reusability and potential applicability for real-time automotive exhaust monitoring. Overall, this demonstration validates the effectiveness and feasibility of CQW-OFLs as optical sensors. With their structural flexibility and high sensitivity, CQW-OFLs offer a strong potential for integration into future real-time automotive exhaust monitoring systems.

## Conclusions

In summary, a CQW-activated multiwavelength laser source has been successfully developed. Through size control, elemental doping, and heterostructure engineering design, we synthesized four types of CQWs with distinct emission wavelengths spanning the green-to-red spectrum. The synthesized CQWs exhibit a high PLQY and low stimulated emission thresholds. Benefiting from their solution processability, the CQWs spontaneously deposit into emitters through capillary action, significantly simplifying the fabrication process. These proposed multiwavelength laser sources based on CQWs have demonstrated excellent laser characteristics, including narrow line width (below 50 pm) and high stability. Furthermore, we have demonstrated the great potential of CQW-OFLs for development in automobile exhaust sensing. These integrated, multiwavelength, high-quality CQW-OFLs are anticipated to play a significant role in the field of optoelectronics, with broad prospects in optical sensing, modern communication, and laser displays.

## Methods

### Preparation of the CQW-Optical Fiber Laser

The emitters used for the CQW-optical fiber lasers consist of commercially available multimode fibers and hollow silica capillaries. The multimode fiber used in this work has a core diameter of 62.5 μm and a cladding diameter of 125 μm. The silica capillary, provided by Polymicro Technologies, features an outer diameter of approximately 150 μm, with the inner diameter selectable from 5 to 50 μm depending on specific experimental requirements. The multimode fibers and silica capillaries are fusion spliced by using a fiber splicer to ensure low-loss and mechanically stable coupling. The silica capillary can be further tailored by using the flame-pulling method to adjust the cavity diameter. To assemble the device, the CQW solution is introduced into the hollow capillary via capillary action. As the solvent evaporates, CQWs spontaneously deposit at the tip of the capillary, forming a compact and localized gain region that serves as the laser emitter. Note that Figure 2a serves as a conceptual illustration for visualization purposes. In practical implementation, each device used in our experiments consists of a single multimode fiber coupled to a silica capillary. Looking ahead, integrating multiwavelength lasers into a fiber bundle via a fiber coupler is entirely feasible and offers promising potential for scalable applications.

## Supplementary Material



## Notes

CQW synthesis; device fabrication; ASE and laser measurement; numerical simulation; SEM of device; TEM of CQWs; laser mode analysis; time-resolved PL decay curves, Auger lifetime; supplementary experiment on exhaust detection (PDF)
