# Supplementary material for: Multiwavelength, Ultranarrow Line Width Emission from Fiber-Capillary-Integrated Colloidal Quantum Well Lasers
Source: ACS Nano. 2025 Jul 21;19(29):26514–24. doi: 10.1021/acsnano.5c04750 (PMC12312156; doi:10.1021/acsnano.5c04750)
Supplement: Supplementary file 1 [file nn5c04750_si_001.pdf]

Supporting Information *for*

Multi-Wavelength, Ultra-Narrow Linewidth Emission from Fiber-Capillary Integrated Colloidal Quantum Well Lasers

Rui Duan[1,], Yi Tian Thung[2], Yichen He[3], Guodan Wei[1], Yuan Wang[1], Lian Xiao[2], Zitong Zhang[4], Tianhua Ren[1], Lin Zhang[3], Van Duong Ta[5,]*, Handong Sun[1,]**

[1]Institute of Applied Physics and Materials Engineering, University of Macau, Macao SAR 999078, China

[2]Division of Physics and Applied Physics, School of Physical and Mathematical Sciences, Nanyang Technological University, 21 Nanyang Link, Singapore 637371, Singapore

[3]School of Precision Instruments and Optoelectronics Engineering, Tianjin University, Tianjin 300072, China

[4] Institute of Semiconductors,Guangdong Academy of Sciences, Guangzhou, Guangdong 510650, China

[5]Department of Optical Devices, Le Quy Don Technical University, Hanoi 100000, Vietnam

* E-mail: ruiduan@um.edu.mo; duong.ta@lqdtu.edu.vn; hdsun@um.edu.mo

Contents

S1. Chemicals S-3

S2. Synthesis of the 4 ML core CdSe CQWs S-3

S3. Synthesis of the 4 ML core CdSeS CQWs S-3

S4. Synthesis of the red-emitting core-shell CdSe/ $Cd_{1-x}Zn_xS$ CQWs S-4

S5. Synthesis of orange-emitting CdSe/$Cd_{1-x}Zn_xS$ core/thin-shell CQWs S-4

S6. Synthesis of yellow-emitting CdSeS/$Cd_xZn_{1-x}S$ core/shell CQWs S-5

S7. Synthesis of green-emitting bromide ligand-capped CdSe CQWs S-5

S8. Measurement of PLQY S-5

S9. SEM and TEM Characterization S-6

S10. ASE spectra measurement S-6

S11. Lasing Mode number analysis S-6

S12. Automobile Exhaust sensing S-7

Figure S1. Bright-field TEM images of various types of CQWs S-8

Figure S2. GSB decay curves for CQWs S-9

Figure S3. Biexciton Auger lifetimes of four CQW types S-10

Figure S4. Schematic diagram of the measurement setup for CQW-OFLs S-11

Figure S5. FWHM of emission peaks as a function of pump power of CQW-OFLs S-12

Figure S6. 3D FDTD simulation structure diagram of CQW-OFLs. S-12

Figure S7. Mode analysis of the lasing spectrum from a CQW-OFL S-13

Figure S8. FSR as a function of the inverse of the diameters of CQW-OFLs S-13

Figure S9. SEM image of a thin silica fiber coupled to a CQW-OFL S-14

Figure S10. SEM image of the tip of a capillary microtube S-14

Figure S11. The time-resolved PL decay curves for FRET system S-15

Figure S12. ASE measurement of the FRET film of CQWs S-16

Figure S13. Comparison of laser intensity at different response times S-17

Figure S14. Spectral response of the CQW-OFL S-17

Table S1. Lasing thresholds of different types of CQW-OFLs S-18

Table S2. Analysis of time-resolved PL decay curves S-18

References S-19

Experimental

S1. Chemicals

All reagents were used as received without further purification. Technical grade 1-octadecene (ODE, 90%), oleic acid (OA, 90%), oleylamine (OLAm, 70%), selenium powder (trace metals basis ≥ 99.99%), Cadmium nitrate tetrahydrate ($Cd(NO_3)_2 \cdot 4H_2O$, 98%), cadmium acetate dihydrate ($Cd(OAc)_2 \cdot 2H_2O$, 98%), myristic acid (≥ 99%), sodium hydroxide (97%), 1-octanethiol (98%), cadmium oxide (CdO, trace metals basis ≥ 99.99%), zinc acetate dihydrate ($Zn(OAc)_2 \cdot 2H_2O$, trace metals basis ≥ 99.99%) were all purchased from Sigma-Aldrich. Acetonitrile (HPLC, ≥ 99.9%) was purchased from J. T. Baker. Hexane and methanol were purchased from Fisher Scientific.

S2. Synthesis of the 4 ML core CdSe CQWs

77 mg of CdO and 340 mg of myristic acid were dissolved in 28 mL of ODE, and the solution was degassed at 100 °C. The temperature was increased to 285 °C under argon flow, with the solution heated until it turned colourless. The solution was then cooled to 90 °C for injection of 24 mg of Se powder dissolved in 2 mL of ODE, and then the temperature was raised to 235 °C. At 195 °C, 160 mg $Cd(OAc)_2{\cdot}2H_2O$ was added swiftly to initiate anisotropic growth. After 10 minutes of growth at 235 °C, the reaction was terminated with the addition of 1 mL OA and was cooled to room temperature by a water bath. The CdSe CQW solution was diluted with the addition of 5 mL of hexane and was washed with the addition of ethanol, followed by centrifugation at 6000 rpm. Precipitates were re-dispersed in hexane for further shell growth.

S3. Synthesis of the 4 ML core CdSeS CQWs

Add 0.34g of Cd myristate and 30 mL of ODE to a three-neck flask, and degas the reaction mixture at room temperature for 20 min. Thereafter, switch to N_2 gas and inject 2 mL of Se-SUS solution, prepared by dissolving 80 mg of Se powder in 8 mL of ODE. Switch to vacuum and degas the reaction mixture at 95 ºC for 20 min. Thereafter, add 0.25 mL of 0.2 M S-ODE solution into the reaction mixture under N_2 gas at 100 ºC. Raise the temperature of the reaction mixture to 230 ºC. When the color of the solution mixture turns bright yellow at around 195 ºC, swiftly add 70 mg of $Cd(Ac)_2$ into the flask. When the temperature reaches 230 ºC, the solution

mixture is annealed for 10 min to allow the CQWs to grow. Thereafter, add 1 mL of OA to the solution and cool it to room temperature using a cold bath. Add 5 mL of hexane to the solution before obtaining the CQWs as the supernatant via selective precipitation. The CQWs are then washed twice with ethanol before dispersed in hexane.

S4. Synthesis of the red-emitting core-shell CdSe/ $Cd_{1-x}Zn_xS$ CQWs

0.1 mmol of $Cd(OAc)_2{\cdot}2H_2O$, 0.3 mmol of $Zn(OAc)_2$, 10 ml of ODE and 1 mL of oleic acid were degassed under vacuum at 90 °C for an hour in a 50 mL three-neck round bottom flask and heated under argon at 200 °C for 15 min. Once the mixture was cooled to 80 °C, 1 mL of the 4-monolayer CdSe core CQWs in hexane and 1 mL of OLA were injected into the solution. Then, the temperature was set to 300 °C under argon. Subsequently, 0.1 M 1-Octanethiol-ODE mixture was prepared in the glove box, and injection of the mixture was started at 163 °C by using a syringe pump with a rate of 5 mL/h until 240°C. After the temperature reached 240 °C, the injection rate was changed to 10 mL/h. The reaction was maintained at 300 °C until all precursors were injected into the flask, and the reaction was cooled down to room temperature by a cold-water bath. The synthesized core-shell CQWs were washed with ethanol by centrifugation at 6000 rpm and redispersed in hexane.

S5. Synthesis of orange-emitting CdSe/$Cd_{1-x}Zn_xS$ core/thin-shell CQWs

Dissolve 0.1 mmol of $Cd(Ac)_2$, 0.3 mmol of $Zn(Ac)_2$, 10 mL of ODE, 1 mL of OA and 1 mL of 4 ML CdSe core CQWs (OD = 1 at 350 nm of its absorption profile) in a three-neck flask. Degas at room temperature for 30 min. Thereafter, raise the temperature to 90 ºC and degas for another 30 min. Switch to N_2 gas, and inject 1 mL of OLAm. Raise the temperature of the reaction mixture to 300 ºC. Mix 140 µL of octanethiol in 5 mL of ODE. When the temperature of the reaction mixture reaches 160 ºC, increase the temperature at a rate of 10 ºC every 3 min and inject the octanethiol solution into the three-neck flask dropwise at a rate of 10 mL/h until 240 ºC, before switching to 5 mL/h and increasing the temperature at a rate of 10 ºC every 2 min. Continue adding the precursor until the temperature of the reaction mixture reaches 300 ºC, where the temperature is held constant until all the precursor has been added. The reaction

mixture is subsequently annealed for 20 min, before terminating the reaction using a cold bath. When the temperature of the solution drops to 50 ºC, 7 mL of hexane is added. Once the solution cools to room temperature, the CQWs are obtained as a supernatant via selective precipitation. It is then washed twice with ethanol before subsequently dispersing in hexane or octane for further use.

S6 Synthesis of yellow-emitting CdSeS/$Cd_xZn_{1-x}S$ core/shell CQWs

Dissolve 0.1 mmol of $Cd(Ac)_2$, 0.3 mmol of $Zn(Ac)_2$, 10 mL of ODE, 1 mL of OA in a three-neck flask. Degas at 130 °C for 20 min. Thereafter, reduce the temperature to 90 ºC and switch to N_2 gas. Inject 1 mL of 4ML CdSeS core CQWs (OD = 1), and degas for another 30 min at 90 ºC. Switch to N_2 gas, and inject 1 mL of OLAm. Raise the temperature of the reaction mixture to 300 ºC at a rate of 15 ºC/min. Mix 140 µL of octanethiol in 5 mL of ODE. When the temperature is raised to 165 ºC, start injecting the octanethiol solution at a rate of 10 mL/h. When the temperature reaches 245 ºC, change the injection rate to 5 mL/h. Hold the temperature at 300 ºC and continue adding the octanethiol solution until finish. Thereafter, anneal the solution for 30 min, before terminating the reaction with a cold bath. When the temperature of the solution drops to 50 ºC, 7 mL of hexane is added. Once the solution cools to room temperature, the CQWs are obtained as a supernatant via selective precipitation. It is then washed twice with ethanol before subsequently dispersing in hexane for further use.

S7. Synthesis of green-emitting bromide ligand-capped CdSe CQWs

Firstly, a bromide solution is prepared by dissolving 0.25 mmol of $Cd(Br)_2 \cdot 4H_2O$ in 5 mL MeOH. Subsequently, 0.2 mL of 4 ML CdSe CQW solution is mixed with 8 mL of toluene and 0.4 mL of OLAm under rapid stirring. 0.6 mL of the as-prepared bromide solution is added gently to the reaction mixture, which is then left to stir continuously for 2 days. Thereafter, the ligand-exchanged CQWs are obtained via selective precipitation, as the precipitate from washing the reaction mixture with 1 mL of methanol at 8500 rpm for 5 min. The CQWs are subsequently dispersed in hexane for further use.

S8. Measurement of Photoluminescence Quantum Yield (PLQY)

The PLQY was measured using a commercial quantum efficiency measurement system, with PLQY defined as the ratio of the number of photons emitted to the number of photons absorbed. Specifically, 1 mL of reference solution (in our case, hexane) was loaded into a cuvette as the reference sample. The number of exciting photons was determined by measuring the excitation light spectrum. Subsequently, the reference solution was replaced with 1 mL of the CQW solution, allowing the acquisition of both the transmitted and fluorescence spectra. The number of absorbed photons was calculated by subtracting the transmitted light spectrum from the excitation light spectrum, while the number of emitted photons was obtained from the fluorescence spectrum.

S9. Scanning Electron Microscope (SEM) and Transmission Electron Microscopy (TEM) Characterization.

For SEM characterization, a FESEM JEOL JSM-7800F PRIME field emission scanning electron microscope was used. This SEM provides high resolution for precise imaging of microstructures and detailed analysis of their topological and morphological features. For TEM characterization, bright-field images of CQWs drop-cast onto 3.05 mm diameter carbon-coated copper grids were obtained using a JEOL JEM-1400 electron microscope operating at 100 kV, enabling characterization of the CQWs' dispersion, morphology, and size.

S10. ASE Spectra Measurement

For ASE spectra measurements, a nanosecond pulsed laser (wavelength: 355 nm, repetition rate: 20 Hz, pulse duration: 5 ns) was employed. Pump-intensity-dependent edge emission was collected via a fiber-coupled spectrometer and detected by silicon charged coupled device (CCD). A Thorlabs DCC1645C USB 2.0 CMOS microscopy camera was used for capturing image of the spot size and subsequent calculations of spot size area for derivations of pump fluences from measured pump intensities.

S11.Lasing Mode Number Analysis

For CQWs-based optics fiber lasers, the lasing modes can be predicted from the following asymptotic equation [1, 2]

$$\lambda^{-1}(D, n_1, n_r, q, m)$$

$$= \frac{1}{\pi D n_1}\left[m + \frac{1}{2} + 2^{-\frac{1}{3}}\alpha(q)\left(m + \frac{1}{2}\right)^{\frac{1}{3}} - \frac{L}{\left(n_r^2 - 1\right)^{\frac{1}{2}}} + \frac{3}{10}2^{-\frac{2}{3}}\alpha^2(q)\left(m + \frac{1}{2}\right)^{-\frac{1}{3}} - 2^{-\frac{1}{3}}L\left(n_r^2 - \frac{2}{3}L^2\right)\frac{\alpha(q)\left(m + \frac{1}{2}\right)^{-\frac{2}{3}}}{\left(n_r^2 - 1\right)^{\frac{3}{2}}}\right]$$

where D is the inner diameter of the capillary tube, m is the mode number, n_1 is the refractive index of the CQWs, n_2 is the refractive index of the SiO_2, $n_r = n_1/ n_2$, $L = 1/n_r$ for transverse magnetic (TM) modes and $L = n_r$ for transverse electric (TE) modes; $\alpha(q)$ expresses the roots of the Airy function, with q as the radial mode number. For first-order radial mode q=1, $\alpha(q)$=2.338, For second-order radial mode q=2, $\alpha(q)$=4.088. After calculating with D = 17 μm, n_1 = 1.87, n_2= 1.45, the lasing peaks fit well with the fundamental TM modes and second-order TE modes, as shown in Figure S7.

S12. Automobile Exhaust sensing

In the experiment, automobile exhaust was collected and subsequently introduced into a sealed chamber through a designated inlet. A CQW-OFL was placed inside the chamber to monitor spectral responses under exposure to automobile exhaust. The laboratory environmental conditions were maintained at a temperature of 22 °C and a relative humidity of ~ 35%. The exhaust was generated from a gasoline-powered passenger vehicle equipped with a turbocharged direct injection engine, operating on premium unleaded gasoline (Research Octane Number, RON 95), which is commonly used in high-compression engines.

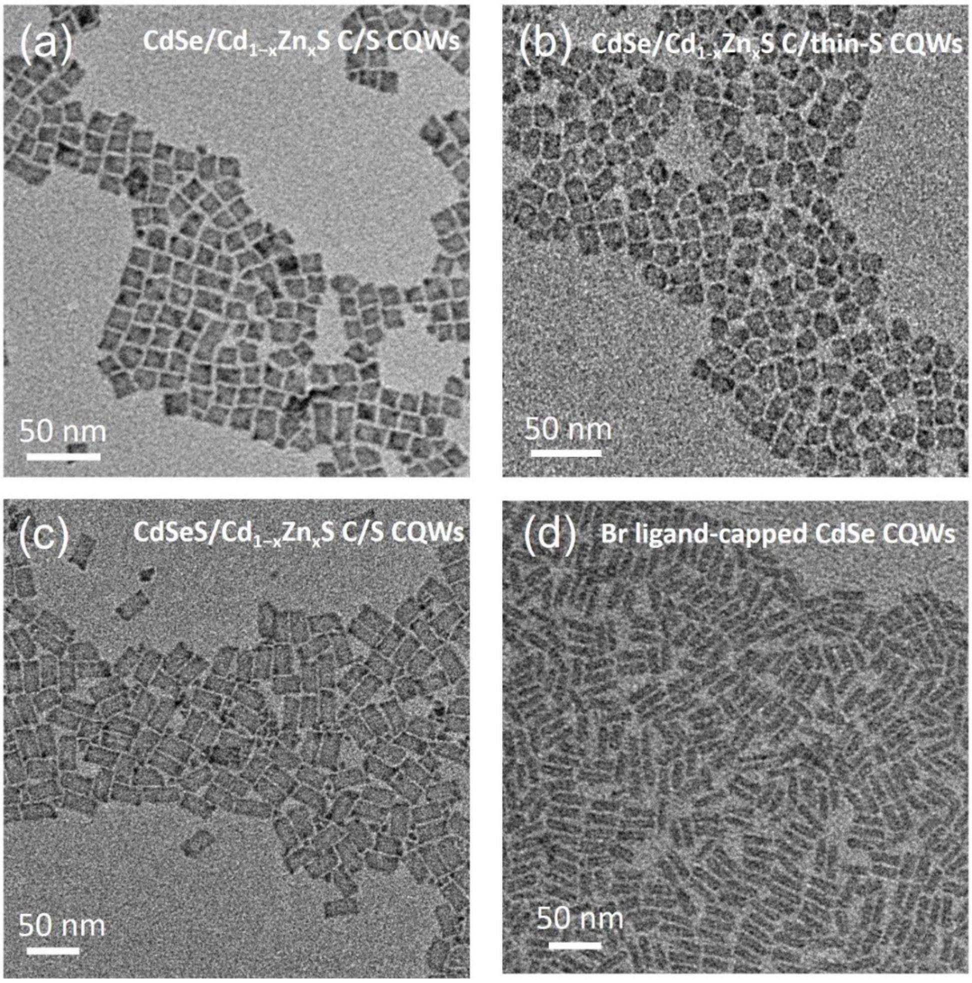

Figure S1. Bright-field TEM images of (a) red-emitting $CdSe/Cd_{1-x}Zn_xS$ core/shell CQWs, (b) orange-emitting $CdSe/Cd_{1-x}Zn_xS$ core/thin-shell CQWs, (c) yellow-emitting $CdSeS/Cd_{1-x}Zn_xS$ core/shell CQWs, and (d) green-emitting bromide ligand-capped CdSe CQWs.

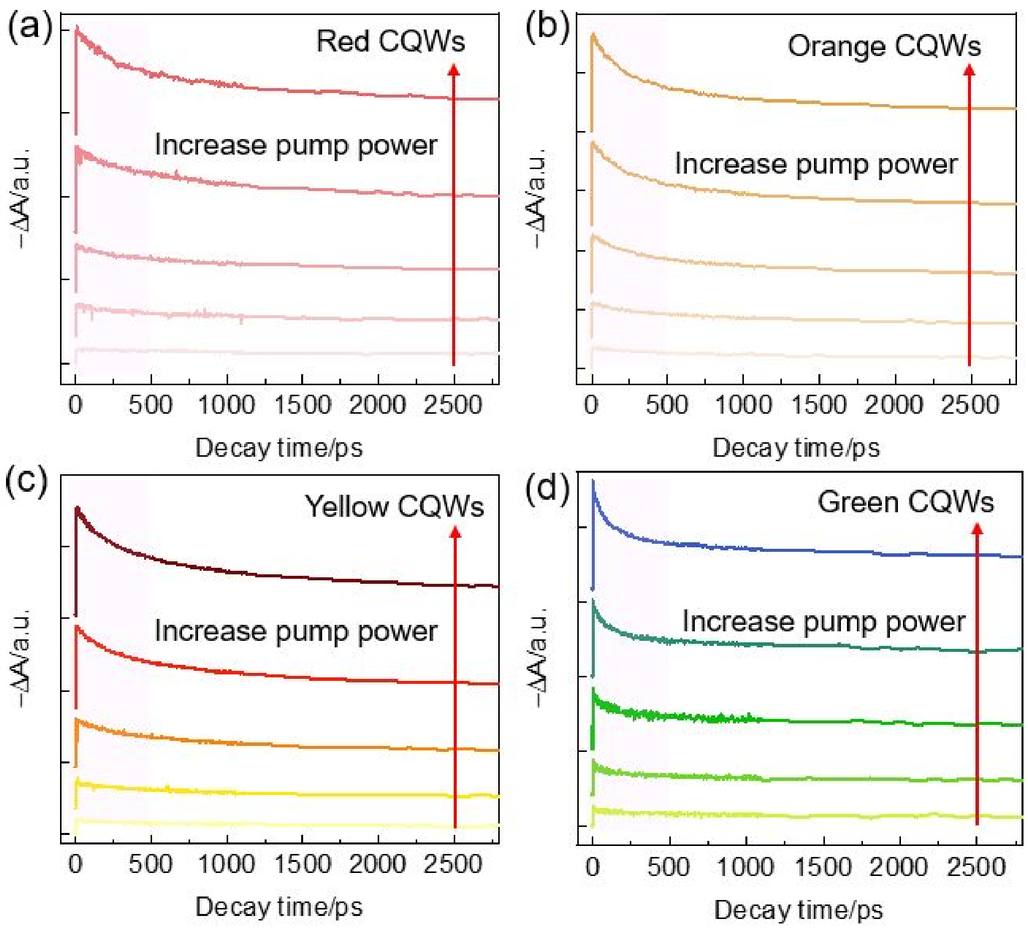

Figure S2. GSB decay curves for (a) CdSe/ $Cd_{1-x}Zn_xS$ core/shell, (b) CdSe/ $Cd_{1-x}Zn_xS$ core/thin-shell, (c) CdSeS/ $Cd_{1-x}Zn_xS$ core/shell, and (d) bromide ligand-capped CdSe CQWs under various excitation intensities. The pump laser is a femtosecond pulsed laser with a 100 fs pulse width at 350 nm and a repetition rate of 1 kHz.

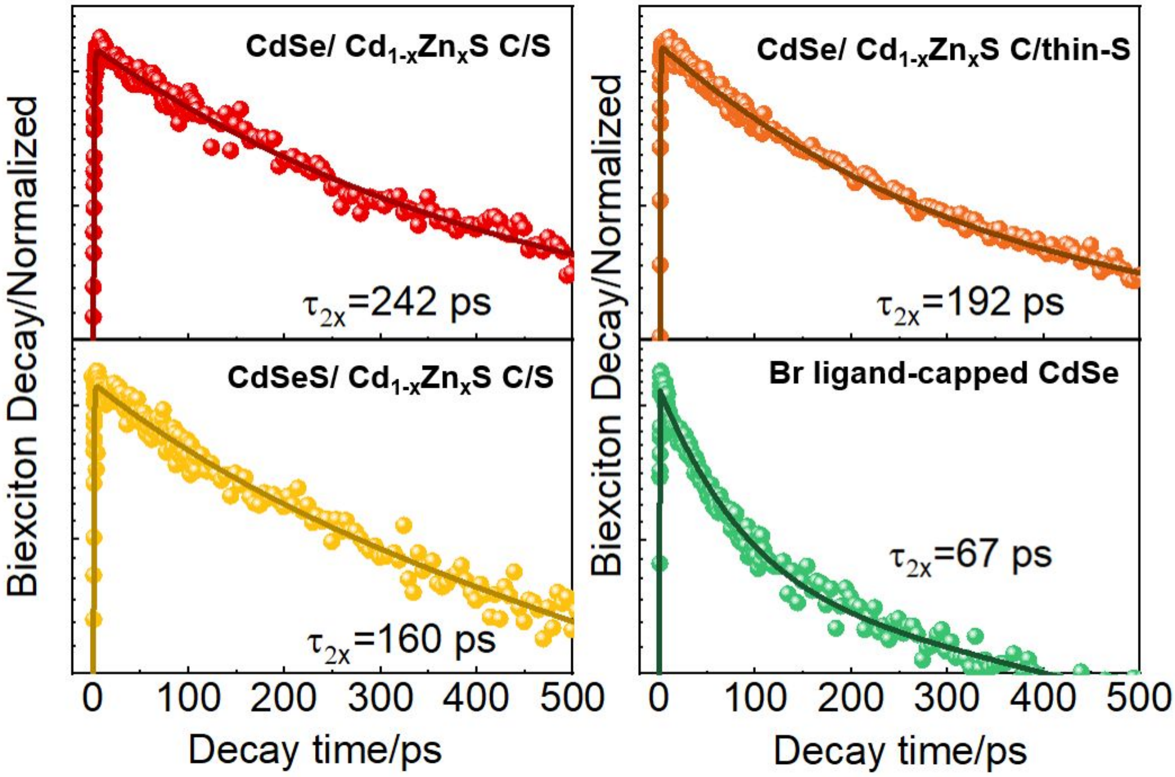

Figure S3. Biexciton Auger lifetimes of four CQW types.

The extracted Auger lifetimes for the four CQWs — CdSe/ $Cd_{1-x}Zn_xS$ core/shell, CdSe/ $Cd_{1-x}Zn_xS$ core/thin-shell, CdSeS/ $Cd_{1-x}Zn_xS$ core/shell, and bromide ligand-capped CdSe CQWs — are 242 ps, 192 ps, 160 ps, and 67 ps, respectively. These lifetimes are significantly shorter than the corresponding single-exciton PL lifetimes (on the nanosecond scale), confirming that the fast component arises from nonradiative Auger recombination[4].

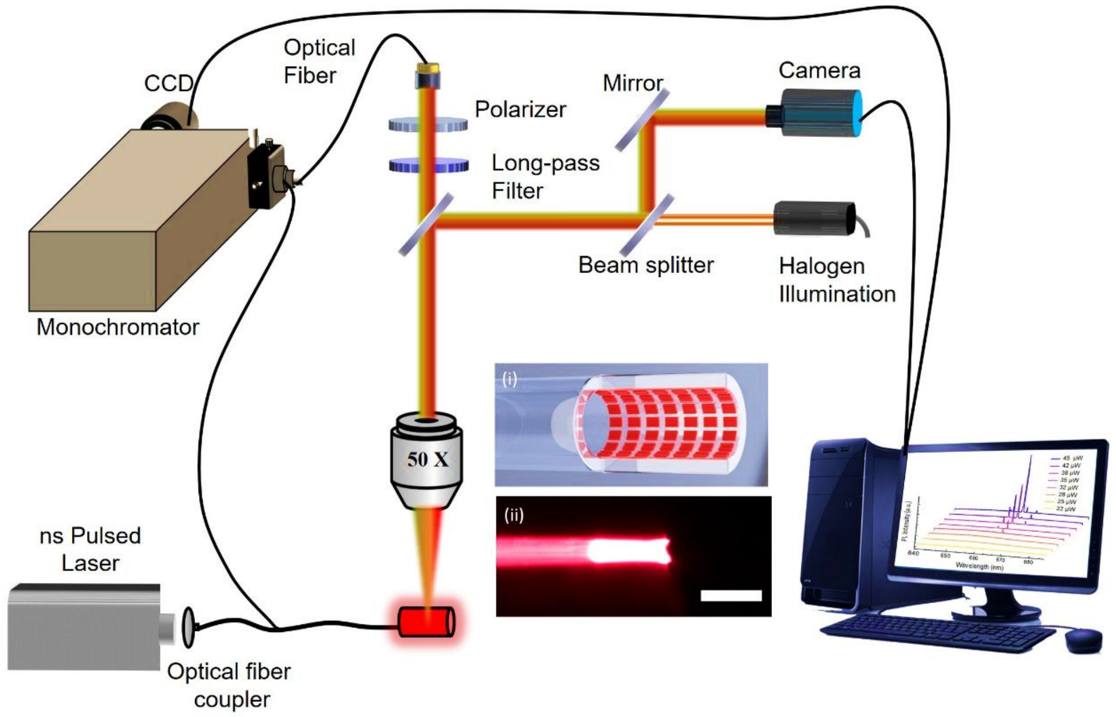

Figure S4. Schematic diagram of the measurement setup for CQW-OFLs. The lasing behavior of CQW-OFLs was investigated using a homemade PL system. A pulsed nanosecond laser (wavelength: 355 nm, repetition rate: 20 Hz, pulse duration: 5 ns) served as the pump source. CQWs self-assemble into laser emitters at the ends of capillary microtubes and are stimulated through fiber coupling, thus eliminating the need for complex micro-focusing systems. The output signal of the CQW-OFLs is connected to a spectrometer equipped with a silicon charge-coupled device (CCD) via an optical fiber coupler for spectral recording (spectral resolution of 0.012/0.043 nm). For image acquisition and PL calibration, a 50× objective lens with a numerical aperture of 0.42 was used to collect the signal from the top of the CQW-OFLs. The captured light signal was either transmitted to a camera for photoluminescence imaging or coupled to the spectrometer.

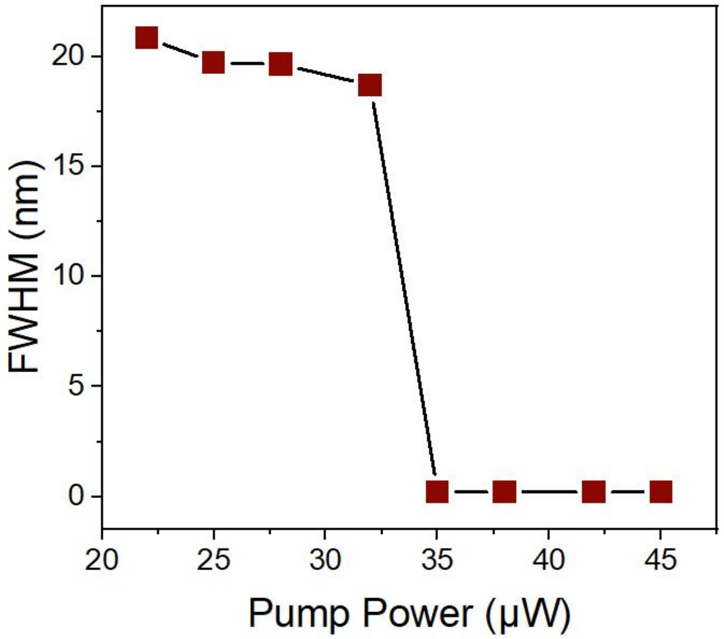

Figure S5. FWHM of emission peaks as a function of pump power of CQW-OFLs with a diameter of ~17 μm.

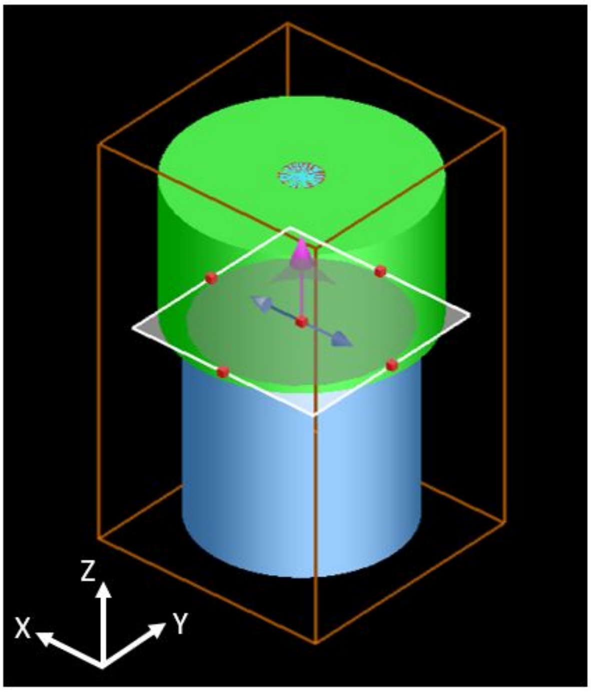

Figure S6. 3D FDTD simulation structure diagram of CQW-OFLs. The blue section represents the multimode fiber, while the green section depicts the capillary microtube carrying CQWs. Due to memory constraints, the dimensions of the laser structure have been scaled proportionally.

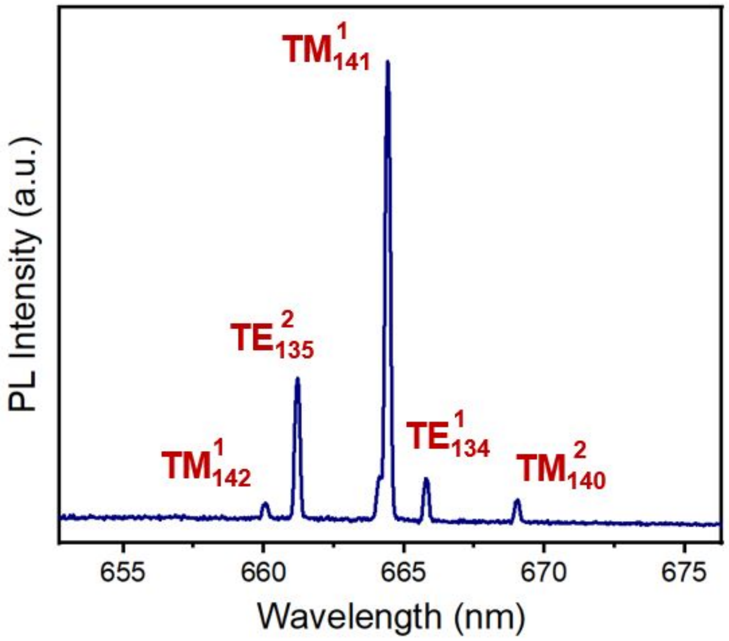

Figure S7. Mode analysis of the lasing spectrum from a CQW-OFL with a 17 μm inner diameter. For detailed analysis, please refer to S11 Mode Number Analysis.

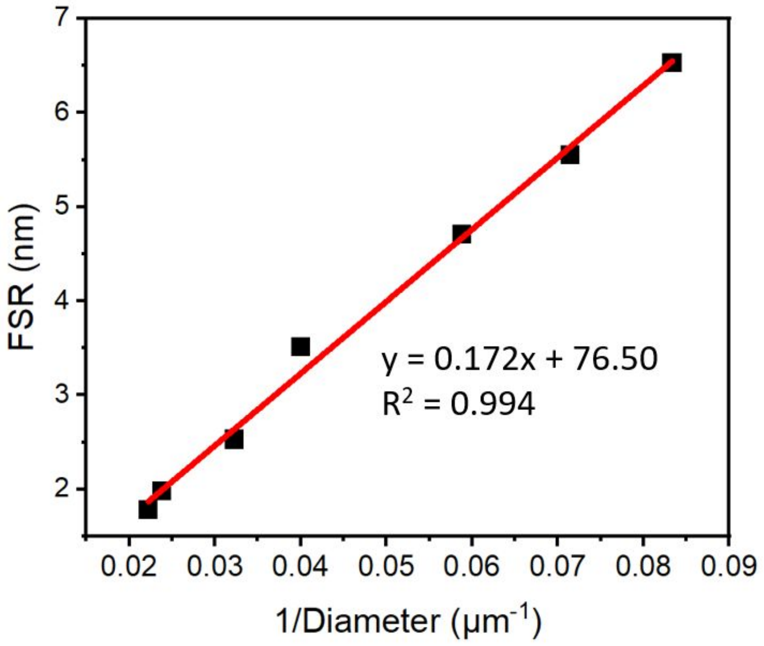

Figure S8. FSR as a function of the inverse of the diameters of CQW-OFLs, demonstrating a linear relationship.

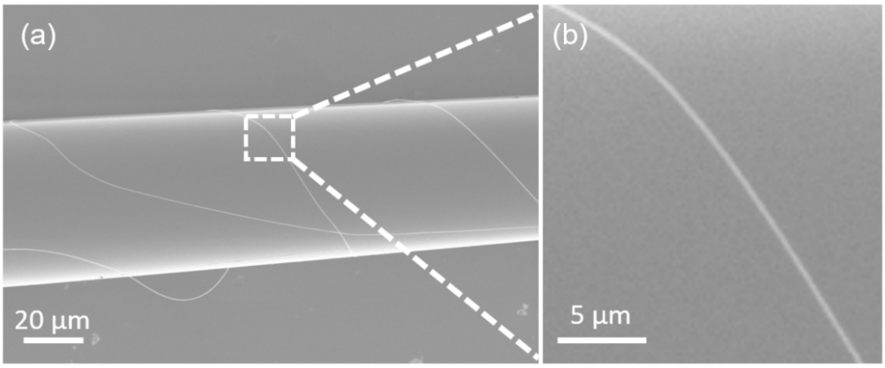

Figure S9. (a) SEM image of an ultrafine silica microfiber waveguide coupled to a CQW-OFL. (b) A magnified view of the corresponding area, indicating that the diameter of the microfiber is ~ 500 nm.

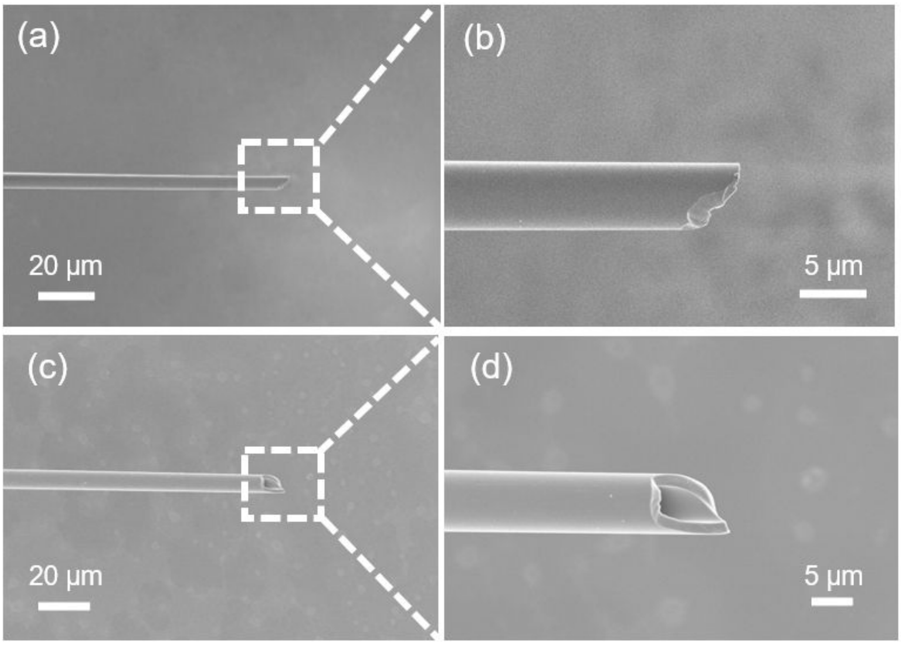

Figure S10. (a, c) SEM image of the tip of a capillary microtube created using the flame pulling method. (b, d) A magnified view of the corresponding area. The tip was left untrimmed to facilitate observation of the microtube's hollow interior.

To justify the occurrence of single-mode lasing, we evaluated the WGM cavity size after flame pulling, where the silica capillary's outer diameter was reduced to 5–8 μm and the inner diameter to below 5 μm. A representative SEM image (Figure S8) shows an inner diameter of 3.9 μm. Using an effective refractive index of 1.76, the calculated free spectral range (FSR) for red, orange, yellow, and green CQW-OFLs is 20.5 nm, 17.8 nm, 16.1 nm, and 13.6 nm, respectively. These FSRs significantly exceed the corresponding ASE linewidths (3.7-5.3 nm), and even the low-energy halves of the PL spectra (9.0-16.5 nm), confirming the plausibility of single-mode operation.

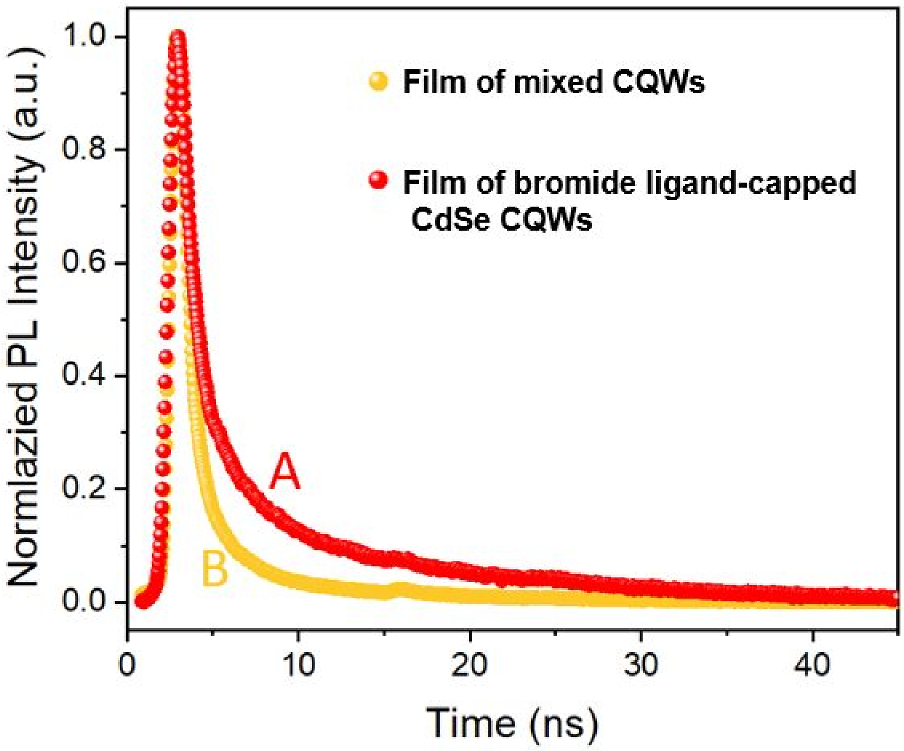

Figure S11. Time-resolved PL decay curves for bromide-ligand-capped CdSe CQWs. The red dots represent the PL decay of the film composed solely of bromide ligand-capped CdSe CQWs (Curve **A**). The yellow dots correspond to the film of mixed CQWs (Curve **B**), consisting of $CdSe/Cd_{1-x}Zn_xS$ core/shell CQWs as acceptors and bromide ligand-capped CdSe CQWs as donors.

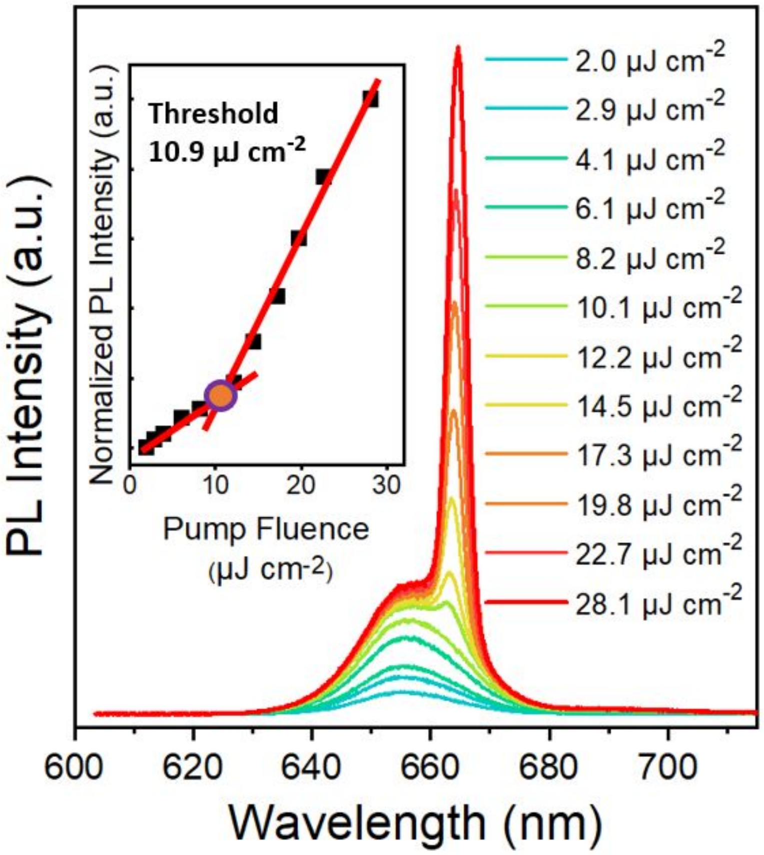

Figure S12. ASE measurement of the film with a specified donor-to-acceptor ratio of CQWs. The ASE threshold was measured to be approximately 10.9 μJ cm^{-2}..

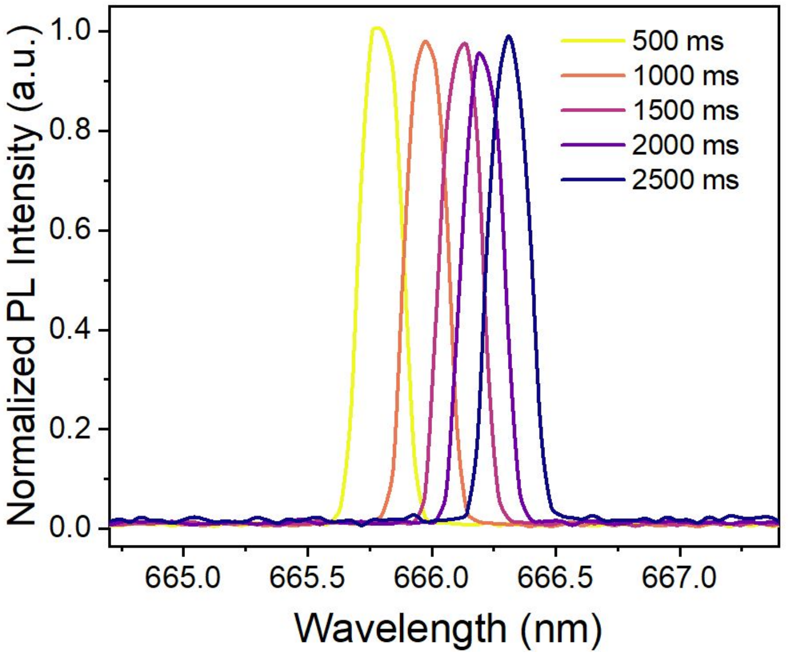

Figure S13. Comparison of laser intensity at different response times.

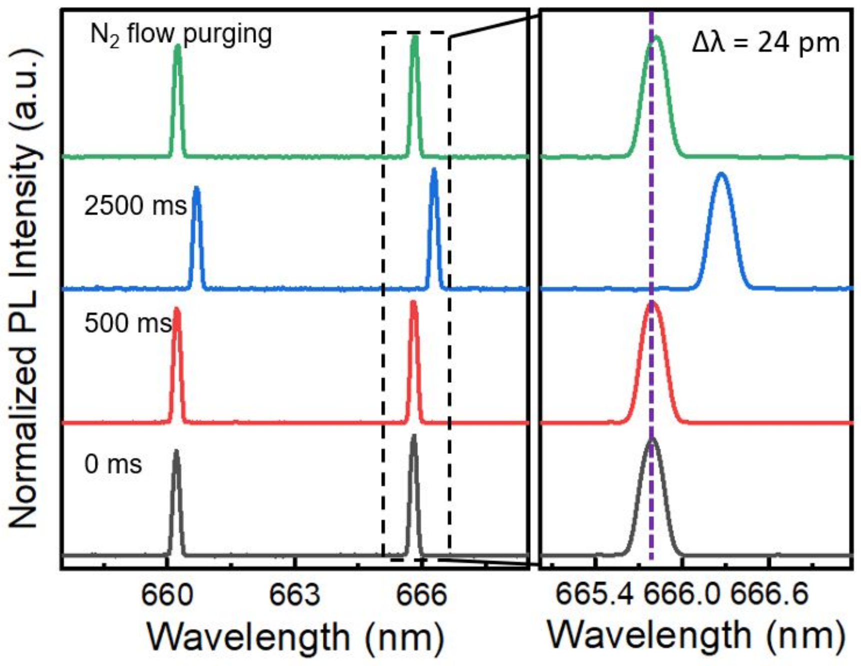

Figure S14. Spectral response of the CQW-OFL to automobile exhaust and spectral recovery after N_2 flow purging. Compared to the initial position, a slight redshift of 24 pm was observed. No significant spectral response was observed at 500 ms due to the instrument response time and delayed automobile exhaust diffusion into the CQW-OFL cavity.

Table S1. Lasing Thresholds of Different Types of CQW-OFLs

Laser Type	Power (μW)	Power density (W cm^{-2})
Multimode red-emission	35	1.14
Sigle mode red-emission	52	1.69
Sigle mode orange -emission	80	2.61
Sigle mode yellow-emission	181	5.90
Sigle mode green-emission	164	5.35
Multimode red-emission under FRET	29	0.95
Multimode green-emission under FRET	256	8.34

Table S2. Analysis of time-resolved PL decay curves for bromide-ligand-capped CdSe CQWs. As shown in Figure S11, the red dots represent the PL decay of the film composed solely of bromide-ligand-capped CdSe CQWs (Curve **A**). The yellow dots correspond to the film composed of mixed CQWs (Curve **B**), consisting of $CdSe/Cd_{1-x}Zn_xS$ core/shell CQWs as acceptors and bromide-ligand-capped CdSe CQWs as donors. The spectra fitted with a two-phase exponential decay function $I(t) = I_0 + A_1 e^{-\frac{t}{\tau_1}} + A_2 e^{-\frac{t}{\tau_2}}$, where $I(t)$ is the intensity measured, I_0 is an offset constant, A_1 and τ_1 are amplitude constant and lifetime of short decay component respectively, and A_2 and τ_2 are amplitude constant and lifetime of long decay component respectively.

Spectra	I_0	A_1	τ_1 (ns)	A_2	τ_2 (ns)	τ_{avg} (ns)[a]
**A**	0.0083	11.23	1.11	0.33	9.82	1.36
**B**	0.0023	0.19	6.22	39.96	0.80	0.83

[a]Average lifetime of bromide ligand-capped CdSe CQWs was calculated using equation: $\tau_{avg} = (A_1\tau_1 + A_2\tau_2) / (A_1 + A_2)$[5]

References

(1) Lam, C. C.; Leung, P. T.; Young, K. Explicit asymptotic formulas for the positions, widths, and strengths of resonances in Mie scattering. *J. Opt. Soc. Am. B* **1992**, 9 (9), 1585-1592.

(2) Yang, S.; Wang, Y.; Sun, H. Advances and Prospects for Whispering Gallery Mode Microcavities. *Adv. Opt. Mater.* **2015**, 3 (9), 1136-1162.

(3) Yoon, D.-E.; Kim, W. D.; Kim, D.; Lee, D.; Koh, S.; Bae, W. K.; Lee, D. C. Origin of Shape-Dependent Fluorescence Polarization from CdSe Nanoplatelets. *J. Phys. Chem. C* **2017**, 121 (44), 24837-24844.

(4) Klimov, V. I. Spectral and Dynamical Properties of Multiexcitons in Semiconductor Nanocrystals. *Annu. Rev. Phys. Chem.* **2007**, 58, 635-673.

(5) Durmusoglu, E. G.; Hu, S.; Hernandez-Martinez, P. L.; Izmir, M.; Shabani, F.; Guo, M.; Gao, H.; Isik, F.; Delikanli, S.; Sharma, V. K.; et al. High External Quantum Efficiency Light-Emitting Diodes Enabled by Advanced Heterostructures of Type-II Nanoplatelets. *ACS Nano* **2023**, 17 (8), 7636-7644.
